# Laparoscopic Cholecystectomy: A New Milestone or a Dangerous Innovation?

**DOI:** 10.1155/1991/35608

**Published:** 1991

**Authors:** John Terblanche

**Affiliations:** 1 ChM FCS (SA) Department of Surgery Medical School University of Cape Town and Groote Schuur Hospital, Observatory Cape Town 7925 South Africa; 2 Co-director Medical Research Council Liver Research Centre University of Cape Town Cape Town South Africa


*HPB Surgery*, 1991, Vol. 3, pp. 177-180
Reprints available directly from the publisher
Photocopying permitted by license only

© 1991 Harwood Academic Publishers GmbH
Printed in the United Kingdom

### LAPAROSCOPIC CHOLECYSTECTOMY: A NEW MILESTONE OR A DANGEROUS INNOVATION?

JOHN TERBLANCHE

*Department of Surgery, University of Cape Town and Groote Schuur Hospital; Co-director, Medical Research Council Liver Research Centre, University of Cape Town, Cape Town, South Africa*

*(Received 3 August 1990)*

KEY WORDS: Laparoscopic surgery: cholecystectomy

Minimal access surgery via the operating laparoscope has been performed by our gynaecological colleagues for many years. General surgeons have been slow to develop expertise in the techniques of laparoscopic surgery. The new small television cameras and the excellent colour television reproduction, together with purpose designed instrumentation, have made safe laparoscopic surgery a reality rather than a dream. The first laparoscopic cholecystectomy was performed in France by Phillippe Mouriat of Lyons in 1987. Francois Dubois of Paris, one of the leading pioneers in the field, performed his first laparoscopic cholecystectomy in May 1988. Several other groups commenced later that year. Eddie Reddick of Nashville, Tennessee and Jacques Perissat of Bordeaux, France, performed their first laparoscopic cholecystectomies in September and November of 1988 respectively[1-4]. These pioneers, as well as others, have developed considerable expertise. By early 1990, Dubois reported 350 cases, Reddick 200 cases, and Perissat 193 patients[1]. Furthermore, many people have received training in laparoscopic cholecystectomy at major institutions. Laparoscopic cholecystectomy appears to have established itself as an important new technique.

Standard open operative cholecystectomy is the "gold standard" for the management of symptomatic gallstones in the gallbladder. It is a little over 100 years since the first successful cholecystectomy was performed by Carl Langenbauch. Operative cholecystectomy has withstood the recent threats of oral drug dissolution therapy and extracorporeal shock wave lithotripsy. Does the advent of laparoscopic cholecystectomy mark a new milestone in management? Alternatively, could this be a dangerous innovation which will lead to morbidity, including common bile duct damage, and life threatening intra-operative haemorrhage requiring rapid laparotomy for control? I believe that laparoscopic cholecystectomy will become a major milestone in general surgery if correctly introduced by adequately trained biliary surgeons with laparoscopic expertise. It is not surprising that the procedure

Address for Correspondence: Professor John Terblanche ChM FCS (SA), Department of Surgery, Medical School, University of Cape Town, Observatory, 7925, CAPE TOWN, SOUTH AFRICA

was the subject of a major symposium at the combined Second World Congress of Endoscopic Surgery and SAGES meeting held in Atlanta, Georgia, in March 1990[1].

The indications for laparoscopic cholecystectomy are the same as for standard operative cholecystectomy, namely, symptomatic gallstone disease with gallstones in the gallbladder. Therefore, it should be considered in patients with biliary colic, those with acute cholecystitis and in patients with gallstone pancreatitis. In the author's view, neither silent gallstones nor biliary "dyspepsia" constitute an indication for standard operative cholecystectomy. Such patients should also not be subjected to laparoscopic cholecystectomy. Inappropriate use, because it is reputedly less traumatic, is totally unacceptable and will bring the procedure into disrepute.

There are clear advantages to laparoscopic cholecystectomy. The procedure abolishes the trauma of access and the transient ileus that occurs after standard operative cholecystectomy. As a result, the hospital stay is significantly shortened and many groups perform the procedure on a day case basis. I believe that it is better for patients to stay in hospital overnight. Because of the short stay, the costs of the cholecystectomy are significantly reduced as are the costs to the labour market because patients can return to work within a week rather than the usual six weeks after standard operative cholecystectomy. A further advantage is the avoidance of a laparotomy scar and the complications associated with a laparotomy. In considering contra-indications, remember that laparoscopic cholecystectomy is a full-scale operation with all the associated dangers of a biliary operation, and that the procedure is performed under general anaesthesia, and takes longer than a standard operative cholecystectomy. The contra-indications are the same as for open operative cholecystectomy and include patients with co-morbid conditions who are unfit for major surgery. In addition, specific contra-indications for the laparoscopic approach include severe peritoneal adhesions and patients with a markedly inflamed gallbladder with a thickened wall where the procedure is likely to be technically difficult. Patients with portal hypertension also constitute a contra-indication. Even open operative cholecystectomy is difficult in patients with portal hypertension and our group have recommended a sub-total cholecystectomy[5]. Many would also consider patients with associated gallstones in the common bile duct as having a contra-indication. It is essential that the patient be fully prepared for open operative cholecystectomy which should be performed immediately if any technical difficulties are encountered, including difficulty in defining the structures in the porta hepatis or if there is haemorrhage which is difficult to control. Haemorrhage can be more difficult to control than at open operation, although local pressure and diathermy usually suffice when the bleeding is from the gallbladder bed. It is also important to remember that depth perception is a problem when using a television screen which presents a two-dimensional image. It is possible to damage the common bile duct while applying a clip to the cystic artery because of inadequate visualisation due to inflammation. One damaged common duct is a major disaster. This again indicates the need for the procedure to be undertaken by surgeons with expertise in biliary surgery.

The percentage of patients who should be converted to formal open cholecystectomy remains unknown, with the reported incidence ranging from 3 to 20 percent[1]. I believe that, if laparoscopic cholecystectomy is performed for the correct indications, in excess of 10 percent of patients will either require a primary open cholecystectomy, or should be converted to an open cholecystectomy.

Several technical questions remain unanswered. These include whether intra-operative lithotripsy should be used to fragment the gallstones so that the fragments can be removed using suction prior to removing the gallbladder. I believe this is unnecessary. Another is how to dissect the gallbladder and deal with the gallbladder bed. Does one need a laser or other expensive equipment, or can standard electrocautery and scissors dissection be undertaken? I believe the latter is adequate. Probably, the most important question is, what should be done about patients with stones in the common bile duct, either discovered before commencing laparoscopic cholecystectomy, or during the procedure when laparoscopic cholangiography reveals unsuspected common bile duct stones? There are many views. I submit that a patient with suspected common bile duct stones should have an ERCP pre-operatively. If stones are detected, either ERCP clearance of the common duct followed by laparoscopic cholecystectomy, or standard operative cholecystectomy with exploration of the common bilt duce are equally acceptable options. When unsuspected gallstones are found in the common bile duct during laparoscopic cholecystectomy, I believe that most patients should be converted to a standard formal open operation with cholecystectomy and exploration of the common bile duct. However, in the subset of patients with a very small gallstone or where the filling defect is equivocal, particularly with a small common bile duct, laparoscopic cholecystectomy should be continued and the patients have their ducts evaluated post-operatively by ERCP. If stones are confirmed, they can be removed via the ERCP route. Many would disagree with these recommendations which require further evaluation.

Vitally important questions are, who should perform laparoscopic cholecystectomy and what training is required? This was addressed at the World Congress Symposium[1]. The clear conclusion was that only fully trained general surgeons with expertise in biliary surgery and an adequate training in laparoscopy, should perform laparoscopic cholecystectomy. There was a difference of opinion about how much training in laparoscopic work was required, with general agreement that at least diagnostic laparoscopic expertise was necessary and that it was important for those commencing laparoscopic cholecystectomy to either attend a course, or observe an expert at a major centre. There is no justification for anyone other than a fully trained general surgeon with adequate expertise in biliary surgery to undertake laparoscopic cholecystectomy. In my view, any inadequately trained person who undertakes this procedure, makes themselves liable to justifiable litigation.

### *References*

1. Terblanche, J., Cuschieri, A., Berci, G., Reddick, E. and Perissat, J. (1990) Gallstones in the gallbladder, in or out and how? A panel presentation. *Surgical Endoscopy,* **4**, 127-140
2. Dubois, F., Icard, P., Berthelot, G. and Leward, H. (1990) Coelioscopic cholecystectomy: preliminary report of 36 cases, *Annals of Surgery*. **211**, 60–62
3. Perissat, J., Collet, D. and Belliard, D.R. (1990) Gallstones: laparoscopic treatment — cholecystectomy, cholecystostomy, and lithotripsy. Our own technique. *Surgical Endoscopy*, **4**, 1–5

4. Reddick, E.J. and Olsen, D. (1989) Laparoscopic laser cholecystectomy. *Surgical Endoscopy*, **3**, 131–133
5. Bornman, P.C. and Terblanche, J. (1985) Sub-total cholecystectomy for the difficult gallbladder in portal hypertension and cholecystitis. *Surgery*, **98**, 1–6

*(Accepted by S. Bengmark 8 August 1990)*

